# Quantitative analysis of preferential utilization of AAV ITR as the packaging terminal signal

**DOI:** 10.3389/fbioe.2023.1327433

**Published:** 2023-12-20

**Authors:** Xin Li, Lohra Mickelle Miller, Matthew Chrzanowski, Jiahe Tian, Martin F. Jarrold, Roland W. Herzog, Weidong Xiao, Benjamin Draper, Junping Zhang

**Affiliations:** ^1^ Herman B Wells Center for Pediatric Research, Indiana University IUSM, Indianapolis, IN, United States; ^2^ Chemistry Department, Indiana University, Bloomington, IN, United States; ^3^ Nikegen, Wynnewood, PA, United States; ^4^ Department of Molecular Biology and Genetics, Cornell University, Ithaca, NY, United States; ^5^ Megadalton Solutions Inc, Bloomington, IN, United States

**Keywords:** adeno-associated virus, invert terminal repeat, small genome, packaging signal, replicative intermediates

## Abstract

Genetic engineering advances have led to recombinant adeno-associated virus (rAAV) becoming an invaluable tool for the development of effective gene therapies. The production of rAAV is susceptible to off-target heterogeneous packaging, the effects of which are still being understood. Here, rAAV vectors with four-genome lengths were produced using both adherent and suspension HEK293 cells to understand the 5′ITR termination. AAV8 vectors were produced from the human FVIII plasmid for a full-length cargo of 4,707 nucleotides with specific truncations, creating smaller genomes. Conventionally, rAAV is characterized by differentiating empty capsids from full capsids, but for this work, that description is incomplete. The small genomes in this study were characterized by charge detection-mass spectrometry (CD-MS). Using CD-MS, packaged genomes in the range conventionally attributed to partials were resolved and quantified. In addition, alkaline gels and qPCR were used to assess the identity of the packaged genomes. Together, these results showed a propensity for unit-length genomes to be encapsidated. Packaged genomes occurred as replication intermediates emanating from the 5′ITR, indicating that HEK293 cells prefer unit-length genomes as opposed to the 5′ITR termination and heterogeneous DNA packaging observed previously from Sf9 cell systems. As both manufacturing processes are used and continually assessed to produce clinical material, such an understanding will benefit rAAV design for basic research and gene therapy.

## 1 Introduction

Recombinant adeno-associated virus (rAAV) therapies have been approved for use against several genetic diseases with four current FDA approvals and hundreds more in clinical trials. Wild-type (wt) AAV is a small 26-nm icosahedral capsid that packages a 4.7-kb ssDNA genome. Packaging constraints limit rAAV to approximately 5.2 kb, with optimal packaging remaining near the wt length of 4.1–4.9 kb and excess DNA truncated by cellular nucleases (Dong et al., 1996; Wu et al., 2010). The ssDNA in rAAV is flanked by two inverted terminal repeats (ITRs) of 145 nucleotides (nts), with packaging originating at the 3′ITR. The current AAV packaging model suggests that AAV DNA packaging into the capsid is tightly coupled to its replication process. A successful packaging process requires the presence of pre-assembled capsids, replicated AAV genomes, and viral Rep proteins (Myers and Carter, 1980). The colocalization of Rep proteins, capsids, and AAV DNA in the nucleoplasm of infected cells has been documented (Hunter and Samulski, 1992). During AAV replication, the N-terminus of the large Rep 78 protein (Alex et al., 2012) binds specifically to the Rep-binding element (RBE) within the ITRs and cleaves the terminal resolution site (trs) (Im and Muzyczka, 1989). This cleavage reaction facilitates self-priming and induces further replication. Ultimately, the ssDNA genome is displaced due to the replication of the complementary strand. The displaced AAV genomes are then translocated into preformed empty capsids (Straus et al., 1976; Myers and Carter, 1980). AAV DNA replicative intermediates are concatemers of AAV genomes in a head-to-head configuration. The most prevalent concatemeric species is a dimer, while tetramer and other multimers are also observed (Bennett et al., 2017). When the genome size is less than half of AAV’s packaging capacity, dimer-size genomes (self-complementary AAV) have been observed (Roli and Russell, 2000).

It is unclear how termination sites are determined whether the genome is terminated at the 5′ITR or at its maximum capacity. A significant diversity has been observed in the packaging of both wtAAV and rAAV vectors. Unit-length genomes have been found co-packaged with partial genomes using Sf9 cells (Barnes et al., 2021a). Incomplete genomes have been packaged in wild-type AAV (wtAAV) and rAAV vectors (Hauswirth and Berns, 1979; Carrell et al., 2021). These findings show that AAV packaging does not always terminate at the 5′ITR and may continue until it achieves the packaging capacity limit. Recently, interest in researching packaging heterogeneity has increased to understand the undesirable side effects of AAV dosing, and such outcomes warrant an in-depth analysis of how ITRs are used for the termination of AAV packaging (Colella et al., 2018).

To further determine how ITRs are utilized for AAV packaging termination, four different-sized genomes of 848 bases, 1,320 bases, 2,981 bases, and 4,707 bases, all derived from the human FVIII protein, were encapsidated in AAV8 using suspension and adherent HEK293 systems. All of the AAV samples were then characterized by qPCR, alkaline gel electrophoresis, and charge detection-mass spectrometry (CD-MS), an emerging high-resolution technique.

CD-MS is a single-particle technique that measures the mass-to-charge (*m/z*) ratio and charge simultaneously to determine mass. This is performed for thousands of AAV particles to gain insight into the packaging of the AAV genome. CD-MS has been used previously to analyze various AAV serotypes and genome truncations, as well as antibody binding to AAV (Barnes et al., 2021b; Barnes et al., 2023; Grande et al., 2023). In this work, high-resolution CD-MS was used to resolve small (>700) nt differences in genome packaging.

## 2 Materials and methods

### 2.1 Cell lines

Adherent HEK293 cells (human embryonic kidney (HEK) cells transformed with DNA from human adenovirus type 5, (CRL-1573, ATCC)) were cultured in DMEM supplemented with 10% fetal bovine serum, 100 μg/mL of penicillin, and 100 units/mL of streptomycin (Invitrogen, Carlsbad, CA) and maintained in a humidified 37°C incubator with 5% CO_2_. Suspension HEK293 cells (Viral Production Cells 2.0, Catalog number A49784) were cultured in the Viral Production Medium (Catalog number A4817901) with 4 mM GlutaMAX Supplement (Catalog number 35050038) in a shaker flask. The shaker flask was incubated at 37°C in an incubator with ≥80% relative humidity and 8% CO_2_ on an orbital shaker platform (Celltron, Catalog number I69222).

### 2.2 Plasmid construction

The constructs used in this study were generated from the pAAV-TTR-coFVIII plasmid encoding human FVIII with a TTR promoter. Its backbone length is 6,982 bases. The total length of the vector genome component was 5,202 bases, including the two ITRs. pAAV-TTR-coFVIII was digested with AccI and Xhol, resulting in a 4.3-kb-long fragment being cut out; the rest of the fragment was ligated by itself using the Quick Ligation Kit (Cat# M2200S). Consequently, a complete single-stranded genome ranging from one ITR to another has a length of 848 nucleotides. Likewise, the 1,320-base, 2,981-base, and 4,707-base sequences were acquired by digestion of the pAAV-TTR-coFVIII plasmid with AccI and SphI, BspEI and Xhol, SphI and XbaI, and self-ligation, respectively. The ligation products were transformed into *E. coli* competent cells, and single colonies in a LB/ampicillin plate were screened for the targeted sequences with Smal digestion and Sanger sequencing.

### 2.3 AAV vector production and purification

AAV8 vectors were produced in adherent HEK293 cells and suspension HEK293 cells in parallel. The pFdelta (encoding adenovirus E4, E2A, and VA), pH28 (encoding Rep and Cap proteins), and vector plasmids were delivered at a ratio of 1:1:1 into the adherent HEK293 cells using PolyJet™ DNA *In Vitro* Transfection Reagent (Catalog number SL100688, SignaGen Laboratories). The same triple plasmids at the equal mole ratio were co-transfected into suspension HEK293 cells via FectoVIR®-AAV Transfection Reagent (Catalog number 76469, Polyplus). AAV8 vectors were harvested at 72 h after transfection. Crude vectors precipitated with PEG8000 were incubated at 4°C overnight. Iodixanol gradient ultracentrifugation was applied to purify crude vectors (Crosson et al., 2018). The iodixanol portion in the AAV vector final product was exchanged with 1 ×PBS buffer with extra 0.2 M of sodium chloride. The AAV vectors were stored at −80°C and thawed at 4°C until use. The AAV vectors were titrated by real-time quantitative PCR (qPCR) assay.

### 2.4 qPCR assay

The titers for all AAV vectors were measured by the qPCR method following the previous protocol (Xu et al., 2020) with a minor modification. 10 µL of rAAV vector was added to 90 µL of 1 U/mL DNase I (Thermo Fisher Scientific, Waltham, Ma) and incubated for 30 min at 37°C. To stop the DNase reaction, 1 µL of 0.5 M EDTA (to a final concentration of 5 mM) was added, and the mixture was subsequently heated for 20 min at 85°C. To lyse the virions, 50 µL of lysis buffer containing 40 mg/mL proteinase K (Thermo Fisher Scientific, Waltham, Ma) was added and incubated at 56°C. After 1 h, the temperature was raised to 95°C for 10 min. The copy numbers of vector genomes released were quantified by real-time PCR and expressed in vector genomes/mL (vg/mL). The specific primers used include 1) targeting GOI: forward—GCACATTTCGTAGAGCGAGTG and reverse—CTCCTGGTGAAGGGGCTTTT; 2) targeting GOI: forward—CACTGCTTAAATACGGACGA and reverse—GATCTGCATGGTGGCATCG; and 3) targeting ampicillin gene: forward—GCTCGTCGTTTGGTATGGCTTC and reverse—GGCCGCAGTGTTATCACTCA. Here, it is emphasized that the length from ITR to the ampicillin gene’s end base is 1,822 bases, making it suitable for assessing whether reverse packaging has occurred.

### 2.5 Alkaline gel electrophoresis

AAV vector preparations were treated with DNase (1 U/mL) to remove unpackaged DNA. After heat inactivation, one-half volume of lysis buffer containing proteinase K (40 μg/mL) was added to denature the viral capsid proteins. Then, one volume of phenol:chloroform:isoamyl alcohol (25:24:1) was added. The sample was vortexed and centrifuged at 16,000 g for 30 min at 4°C to remove debris. The supernatant was carefully transferred to a fresh tube. 200 μL of 70% ethanol was added, and then, the sample was centrifuged at 16,000 g for 10 min at 4 °C. The pellet was collected and air-dried at room temperature. The DNA was dissolved in 20 µL of TE buffer. The DNA concentration was measured using NanoDrop One. The extracted DNA was run on an alkaline agarose gel.

### 2.6 CD-MS analysis

All samples (20 µL) were thawed at 4°C and incubated with 1 U/mL of DNase I for 30 min at 37°C before buffer exchange into 200 mM of ammonium acetate (Invitrogen, AM9070G) using desalting spin columns (Bio-Rad, 7,326,228). Ions were generated in positive-mode electrospray ionization using the TriVersa NanoMate (Advion). The CD-MS instrument (Megadalton Solutions) has components described previously (Draper et al., 2018; Hogan and Jarrold, 2018; Todd et al., 2020; Todd and Jarrold, 2020). All experiments, except for the 848-base genome, were run with instrument settings for 130 eV/z ions trapped for 104.6 ms to give charge uncertainty of ∼1 e for an expected mass resolution of 100. To resolve the 848-genome, 400 ms trapping was used to give a charge uncertainty of ∼0.*5 e*. Each data set contained more than 10,000 AAV ions and took between 10 and 52 min, with the higher resolution mode taking a longer time.

### 2.7 Statistical analysis

All data were presented as mean ± SD. Statistical analysis was performed by Student’s unpaired t-test in *SPSS* software version 1.0.0.1406. A *p*-value <0.05 was considered statistically significant.

## 3 Results

### 3.1 Small genomes encapsidated in monomeric or multimeric configurations

**FIGURE 1:**
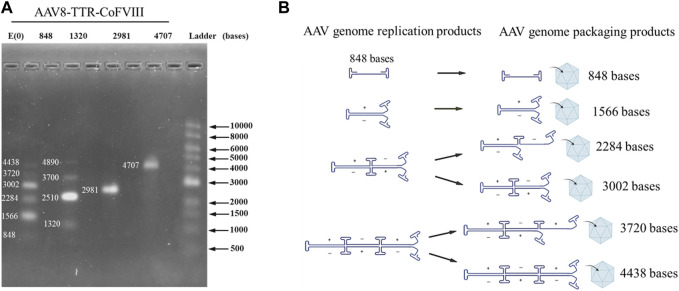
Packaged AAV genome size and molecular configuration. **(A)** The packaged AAV genome size distribution from adherent HEK293 cells analyzed by alkaline gel electrophoresis. **(B)** The package genome molecular configuration model.

To confirm how small genomes package in various AAV populations, viral genomes were extracted and analyzed using alkaline gels. Gels for the AAV8-TTR-coFVIII-2981 and AAV8-TTR-coFVIII-4707 both showed a single band that was assigned to the expected nucleotide length. The AAV8-TTR-coFVIII-848 and AAV8-TTR-coFVIII-1320 also showed the expected gene of interest length (GOI) but also had bands of larger lengths appearing to be multimeric configurations (Figure 1A and Supplementary Figure S1). The vector DNA of AAV-TTR-coFVIII-848 and AAV-TTR-coFVIII-1320 were extracted and digested with Smal restriction enzyme. The resulting digestion product was subjected to alkaline gel analysis. As shown in Supplementary Figure S2, only unit-length genome size bands were observed, suggesting that the DNA molecules with a larger size consist of multiple unit-length genomes. To identify whether the larger-sized DNA band consists of a backbone sequence, two pairs of primers targeting different GOI regions and one pair of plasmid backbone-specific primers targeting the ampicillin gene were used to measure the vector titers. The total length from one ITR to the ampicillin gene’s end base is 1,822 bases, which is suitable for detecting if reverse packaging occurs. As shown in Supplementary Figure S3, there is no significant difference in the ratios of the vector titers between the internal primer group relative to the plasmid backbone primer group among the four vectors. This suggests that the packaged DNA mainly originated from the GOI, without increasing in plasmid backbone packaging. The multimeric nature of the bands points to the encapsidation of replication intermediates. Exact nucleotide assignment is difficult using alkaline gels, but due to the quantized nature of these bands, a replication model was proposed (Figure 1B). The proposed model (Figure 1B) shows self-complementarity occurring at each ITR until the packaging capacity is reached. Packaged nucleotide lengths follow Eq. (1), where N is the number of ITRs, I is the length of the ITR, and G represents the gene length.

Equation 1:Packaged Nucleotides=NI+N−1G.

Using this equation, possible genome lengths for AAV8-TTR-848 are assigned in Figure 1A at nucleotide lengths 848 (1GOI-2ITR), 1,566 (2GOI-3ITR), 2,284 (3GOI-4ITR), 3,002 (4GOI-5ITR), 3,720 (5GOI-6ITR), and 4,438 (6GOI-7ITR). Band intensity indicated that even multimers have higher intensities compared to those at odd multimer locations, meaning complete self-complimentary strands are preferred. These results demonstrate that small genomes were packaged in AAV capsids in monomeric or multimeric molecular forms.

### 3.2 Mass distributions of rAAV population packaging small genomes

**FIGURE 2:**
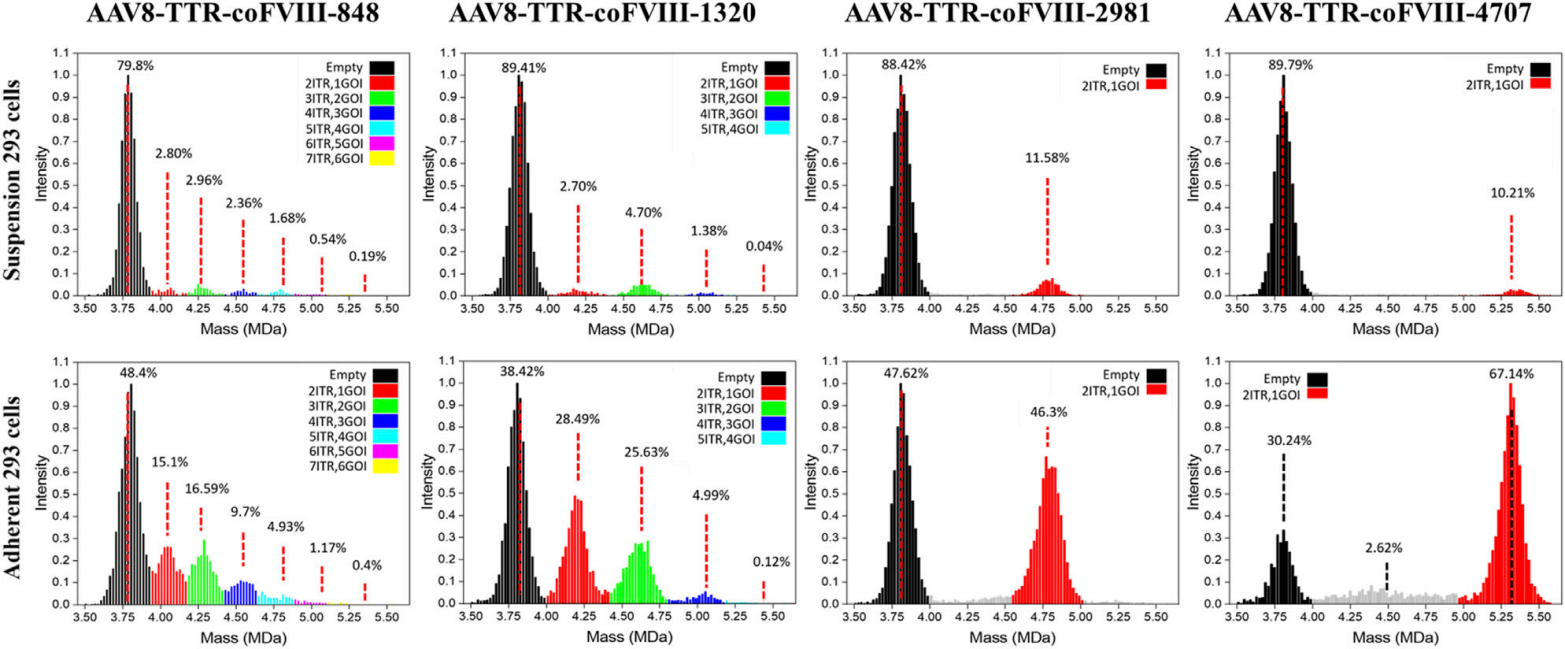
Relative abundance of individual subpopulations in rAAV vector particles. The particle population distribution and their relative abundance analyzed by CD-MS. Top panel: rAAV vectors derived from suspension HEK293 cells. Bottom panel: rAAV vectors derived from adherent HEK293 cells. Packaged nucleotide lengths are labeled with the ITR number.

Mass distributions of the rAAV population and their relative abundances measured for the two cell production systems showed good agreement between the genome packaging species observed in adherent and suspension cell lines, but the adherent production showed significantly more packaged genomes (Figure 2). Due to the small length of the vectors used here, empty and full characterization is not sufficient for the packaging behavior observed. For each plot in Figure 2, the range shown in black represents the empty AAV8 capsid, and the red range represents a single ssDNA (1GOI-2ITR) packaged. The green (2GOI-3ITR), blue (3GOI-4ITR), cyan (4GOI-5ITR), magenta (5GOI-6ITR), and yellow (6GOI-7ITR) quantized gene of interest additions, as shown by the proposed model in Figure 1B. The relative abundance of their individual subpopulations is also listed in Figure 2. The AAV8-TTR-2981 and AAV8-TTR-4704 show one typical empty particle peak and a single clean GOI peak with low abundance partials (gray regions). Meanwhile, it is also shown that the empty particle ratios of the above two vectors increase by 1.86- and 2.97-fold, while their corresponding full particle ratios decrease by 3.99- and 6.58-fold between the suspension *versus* adherent cell system. The vector populations of AAV8-TTR-848 and AAV8-TTR-1320 contain multiple peaks. In the whole AAV8-TTR-848 vector population from suspension HEK293 cells, there are seven peaks, and their relative abundances are 79.8%, 2.8%, 2.96%, 2.36%, 1.68%, 0.54%, and 0.19%, respectively. Among the seven peaks, the highest one indicates an empty particle population, and the other peaks imply vector populations with varying sizes of genomes. The relative abundance of these subpopulations’ packaging DNA was further compared with their corresponding DNA band intensity by alkaline gel analysis, and the two distribution profiles show a consistent trend (Supplementary Figure S4). These results demonstrate that small genomes were encapsidated in various populations.

### 3.3 Preferential utilization of AAV ITR as the packaging terminal signal

Building on the relative abundance of individual subpopulations determined by CD-MS and the relative intensity of DNA bands observed in alkaline gel electrophoresis, we further conducted a quantitative analysis to assess the likelihood that each ITR serves as a termination signal for packaging. The percentages of each subpopulation are relative to the total abundance of all populations and each DNA band in relation to the total intensity of all DNA bands (Supplementary Figure S5). When the small AAV genome is 848 bases long, the AAV capsid has the capacity to package six different sizes of genomes (848, 1,566, 2,284, 3,002, 3,720, and 4,438 bases). According to the CD-MS data, the percentages of packaging termination at each sequential ITR from vectors packaged in suspension cells were as follows: 26.6% (first ITR), 28.1% (second ITR), 22.4% (third ITR), 16.0% (fourth ITR), 5.1% (fifth ITR), and 1.8% (sixth ITR). The alkaline gel analysis yielded termination probabilities of 5.2%, 35.4%, 24.1%, 25.6%, 6.2%, and 3.6% at the first to sixth ITR, respectively. In an alternative scenario with a small AAV genome of 1,320 bases, the AAV capsid may package four different sizes of genomes (1,320, 2,510, 3,700, and 4,890 bases). The termination probabilities from CD-MS analysis were 30.6%, 53.3%, 15.7%, and 0.45% at the first to fourth ITR, respectively. The alkaline gel analysis showed termination probabilities of 14.3%, 45.2%, 25.5%, and 14.9% at the first to fourth ITR, respectively. Similar results from vector preparations in adherent cells were observed (Supplementary Figure S5). These findings suggest that genome packaging may terminate at any ITR, yet packaging small genomes appears more efficient at avoiding AAV overload while also completing packaging faster by terminating at a closer ITR.

## 4 Discussion

The process by which viruses translocate their genomes into empty capsids remains elusive. The current model proposes a tightly coupled replication and packaging process for AAV genomes with the help of AAV Rep proteins, which is essential for successful AAV encapsidation. It suggested that both host and viral proteins bind to replicated AAV genomes, forming complexes with AAV capsids (Dubielzig et al., 1999; King et al., 2001; Yoon-Robarts et al., 2004). Three potential pathways for genome packaging were proposed (King et al., 2001). First, after complete displacement of a single-stranded genome, the DNA is translocated into the capsid in a 3′ to 5′ direction (Dubielzig et al., 1999). Second, a double-stranded dimer or multimer genome is unwinded on the capsid surface while simultaneously inserting the 3′end of one of the single strands into the capsid. Third, the packaging of a double-stranded monomer genome while undergoing simultaneous replication could lead to premature strand displacement. In the second and third scenarios, the involvement of replicative intermediates implies that dimer and multimer genomes can act as packaging substrates, necessitating termination of the packaging reaction. This is likely facilitated by the nicking activity of the Rep 78/68 proteins (King et al., 2001). To date, only dimer intermediates, like self-complementary genome packaging, have been observed in AAV capsids. Larger multimers remain unobserved. According to the encapsidation model proposed above, if a multimer serves as the packaging substrates, the packaged genomes may consist of an even or odd number of unit-length genomes, as these are the displaced strands from the replicative intermediate templates during replication, unless premature termination occurs. To investigate the packaging behavior of larger multimers, we constructed AAV vectors with small genomes so that their replicative intermediates, such as tetramers or even large multimers, fall within the capacity limit.

In this study, we found that small genomes were packaged in various AAV populations with different masses. The packaged DNA showed monomeric, dimeric, and multimeric size distributions and consisted of an odd and even number of unit-length genomes. The bands observed in agarose gel electrophoresis are often not precisely aligned with molecular weights, particularly in the case of fragments containing AAV ITR. However, they indeed show consistency with CDMS data (Supplementary Figure S4, S6). To better understand this packaging process, based on the point that the packaged multimeric DNA resulted from replication intermediates, we proposed a replication model (Figure 1B). We believe the replication model to be the more likely because, during packaging, the genes are unwound as single-stranded DNA forms, packaged, and terminated at the ITR. There have been no conclusive reports of more than a single full-length ssDNA being packaged in AAV. We speculate that in the replication model, even-number unit-length genomes are easier to package due to symmetrical structures with balanced charges compared to odd-number unit-length genomes (Figure 1). Ultimately, a higher-resolution technique was required to confirm the model and assign correct lengths to the packaged genome, necessitating the need for high-resolution CD-MS. It is also the first time to identify the presence of odd multimer genomes and show the evidence of the AAV genome packaged in a single-stranded DNA form and possible premature termination. These findings further support the hypothesis that replicative intermediates like dimer and multimer can serve as packaging substrates.

In theory, the longer the genomes, the lower the opportunity to be captured by capsids; however, we found that the relative abundances of populations containing a single vector DNA did not decrease with an increase in mass or DNA length for all genome sequences. The relative abundance of the green range for the 2GOI-3ITR of the AAV8-TTR-1320 vector is 1.74 greater than that of the red range for the 1GOI-2ITR (Figure 2). This result agrees with data from the gels of the even-numbered multimers having higher intensity. We observed variability in CDMS data across the samples (Figure 2). As alkaline gel results served as the gold standard for confirming the DNA status within the capsid, our conclusions are primarily drawn from the alkaline gel data, with CDMS data providing additional supporting evidence. However, it is important to note that variations in CDMS will not alter the conclusion regarding DNA packaging inside the capsids. A comparison of the masses measured using CD-MS with the masses predicted by the packaging model proposed in Figure 1B shows that the measured masses are around 4% larger than predicted (Supplementary Figure S6, Supplementary Table S1). The extra mass is attributed to counterions and has been observed in previous studies of AAV genome packaging (Barnes et al., 2021a).

For both examples, the replication intermediates would fall within the expected packaging limit of∼ 5.2 kb. The charge measured by CD-MS can be used to infer information about the structure of the ions. If the DNA was not fully packaged, the ions would be expected to have an elevated charge. The charges for all of the genome lengths were similar (despite having different masses due to their different genome lengths). This indicates that the genomes were fully encapsidated (Supplementary Figure S7).

Given the absence of genome truncations in either CD-MS or alkaline gel analysis, we believe that AAV packaging in HEK293 cells prefers unit-length genomes. This differs from findings reported in Sf9 cells (Barnes et al., 2021a). It explains why rBV/Sf9-produced vector genomes are more heterogenous with less well-defined standard genomes than those produced by HEK293 cells (Tran et al., 2022). This study provides the first identification of the presence of odd multimer genomes. In this study, adherent HEK293 cells were shown to be 2x more efficient in packaging DNA than suspension HEK293 cells (Figure 2). It is not expected that cell lines should have such a significant effect on packaging, and we suspect that other factors in the triple transfection process, like transfection reagents and plasmid-to-transfection reagent ratio, were the primary culprits. It should be noted that all empty capsids had masses in line with the 5:5:50 viral protein ratio, indicating that plasmid stoichiometry was in good order. It is still reasonable that cell-related intrinsic mechanisms of transfection dictate AAV packaging behavior. Our findings provide new insights into AAV replication and packaging, which will benefit rAAV design for basic research and gene therapy (Zoratto et al., 2021).

## Data Availability

The datasets presented in this study can be found in online repositories. The names of the repository/repositories and accession number(s) can be found in the article/Supplementary Material.
