# Supplementary material for: Quantitative analysis of preferential utilization of AAV ITR as the packaging terminal signal
Source: Front Bioeng Biotechnol. 2023 Dec 20;11:1327433. doi: 10.3389/fbioe.2023.1327433 (PMC10761532; doi:10.3389/fbioe.2023.1327433)
Supplement: Supplementary file 1 [file Table1.pdf]

Supplementary Table 1 Expected molecular weight of potential genomes

Name	Nucleotide length (bases)	Expected genome size of unit-length genomes (bases)	Expected AAV vector mass of unit-length genomes (MDa)	Measured molecular weight (MDa)
**AAV8-Empty**	**0**	**0**	**~3.718**	**3.80**
**AAV8-TTR-coFVIII-848**	**848**	**848, 1566, 2284, 3002, 3720, 4438, 5156**	**3.98, 4.20, 4.42, 4.64, 4.87, 5.08, 5.30**	**4.02, 4.25, 4.51, 4.75**
**AAV8-TTR-coFVIII-1320**	**1320**	**1320, 2510, 3700, 4890, ~5200**	**4.13, 4.49, 4.86, 5.23, 5.30**	**4.20, 4.61, 5.04**
**AAV8-TTR-coFVIII-2981**	**2981**	**2981, ~5200**	**4.63, 5.30**	**4.79**
**AAV8-TTR-coFVIII-4707**	**4707**	**4707, ~5200**	**5.15, 5.30**	**5.31**

Based on AAV packaging capacity of around 5.2kb, expected genomes include multimers of the nucleotide length, even number unit genomes, odd number unit genomes, and unit genome plus incomplete genome.

M.W. of ssDNA = Calculated using exact sequence with OligoCalc

TAAV8 Capsid M.W.=~3.718 MDa.[28]

Based on the following M_W: VP1 81 kDa; VP2 65 kDa; VP3 60 kDa. VP1:VP2:VP3=1:1:10
